# Giant In-Plane Shrinkage Induced by Structural Phase Transformation in TbCoSi_2_

**DOI:** 10.3390/ma18215064

**Published:** 2025-11-06

**Authors:** Lulu Liu, Dinghui Wang, Shoutao Zhang

**Affiliations:** 1 School of Electronic Engineering, Nanjing Xiaozhuang University, Nanjing 211171, China; 2 National Laboratory of Solid State Microstructures & Collaborative Innovation Center of Advanced Microstructures, School of Physics, Nanjing University, Nanjing 210093, China; 3 Jiangsu Physical Science Research Center, Nanjing 210093, China; 4 School of Materials Science and Physics, China University of Mining and Technology, Xuzhou 221116, China; wangdh@cumt.edu.cn; 5 Key Laboratory of UV-Emitting Materials and Technology of Ministry of Education, School of Physics, Northeast Normal University, Changchun 130024, China; zhangst966@nenu.edu.cn

**Keywords:** metal-based materials, negative thermal expansion, structural phase transition, antiferromagnetic ordering

## Abstract

Metal-based materials, pivotal for industrialization and technological progress, confront the long-standing issue of high thermal expansion, which limits their application in advanced scenarios. With a century-long research history, negative thermal expansion materials, particularly those in intermetallic compounds, offer promising solutions for regulating thermal expansion. Here, we investigate polycrystalline TbCoSi_2_ ingots, revealing a notable 3% in-plane shrinkage from 223 K to 298 K induced by structural phase transitions. Temperature-dependent XRD and Rietveld refinement identify a low-temperature Pbcm space group structure, and the drastic a-axis shrinkage during the phase transition drives the in-plane contraction. Macroscopic magnetic measurements and first-principles calculations reveal an antiferromagnetic structure below 13.7 K, with magnetic and structural phase transitions being independent. These findings present a metal-based weakly magnetic material for precise thermal expansion control, particularly in the uniaxial direction.

## 1. Introduction

The discovery and extensive application of metal-based materials have exerted an irreplaceable and profound influence on the advancement of industrialization, modernization processes, and the evolution of science and technology across multiple fields. Structural materials represented by steel, which possess excellent mechanical strength, ductility, and processability, have become the fundamental building blocks for constructing infrastructure, automotive components, and aerospace structures. Nevertheless, regardless of whether serving as structural or functional materials, metal-based materials have long faced the challenge of high thermal expansion, which leads to thermal expansion mismatch in devices [1,2,3,4]. Specifically, the coefficient of thermal expansion (CTE) of iron is approximately 12 ppm/K, which restricts its application in advanced scenarios that require low dimensional temperature sensitivity and minimal thermal mismatch when assembled with different materials [5]. Consequently, the precise regulation of thermal expansion behavior in metal-based materials has long been recognized as a critical subject meriting in-depth scientific investigation and technological development [6,7,8,9,10].

Negative thermal expansion (NTE) materials have garnered substantial research attention due to their ability to compensate for and regulate thermal expansion, along with their intriguing physical characteristics that involve complex lattice dynamics [11,12]. The research history of NTE materials dates back over a century, with early studies focusing on Invar alloy Fe_65_Ni_35_ [13]. Typically, low thermal expansion (LTE) or even NTE behaviors are exclusively observed in a limited set of materials. These notably include fluoride compounds, oxide compounds such as ZrW_2_O_8_ and Cu_2_P_2_O_7_, anti-perovskite phases like Mn_3_GaN and Mn_3_CuN, as well as a small number of intermetallic compounds and solid solutions [14,15,16,17,18]. In particular, in metal-based materials, NTE phenomena induced by magnetovolume effects, martensitic phase transitions, and valence state transitions have been observed in specific compounds [19,20,21,22,23,24,25], such as La(Fe,Co,Si)_13_, (Zr,Nb)Fe_2_, (Hf,Ta)Fe_2_, MnCoGe, and YbAl_2_. The NTE of these materials plays a crucial role in regulating the CTE by effectively compensating for the thermal expansion of the matrix materials. Nevertheless, inherent limitations in key aspects, such as insufficient shrinkage rate and narrow operating temperature range, frequently render these materials unable to meet the demands of practical applications. Thus, investigating and exploring novel materials for regulating the thermal expansion of metal-based materials remains of significant scientific and practical value.

Metal-based NTE materials occupy a pivotal position in sectors ranging from aerospace engineering to advanced electronic packaging technologies, owing to their inherent advantages in thermal conductivity and mechanical performance [26]. Among them, metallic systems exhibiting NTE driven by magnetovolume effect—such as La(Fe,Si)_13_-based and MnCoGe-based alloys—have attracted extensive attention [24,27]. However, the pronounced magnetism and intense stray magnetic fields inherent to these materials impose significant constraints on their practical applicability. This critical limitation has thus galvanized sustained research efforts toward the development of weakly magnetic metal-based NTE systems, which hold promise for overcoming such bottlenecks and expanding the technological utility of this class of materials. In this context, ternary intermetallic compounds with a 1:1:2 stoichiometry, denoted as HRE–TM–X (where HRE = heavy rare-earth element, TM = transition metal, and X = p-block element), present a fertile ground for discovery due to their diverse crystal structures and tunable magnetic configurations [28,29,30,31,32]. Among them, TbCoSi_2_ stands out as a model system with a magnetically adjustable framework, offering an ideal platform to investigate the interplay between crystal symmetry, magnetic ordering, and anisotropic lattice contraction in metallic NTE materials [33,34,35].

In this work, we investigated the remarkable in-plane shrinkage of up to 3% in polycrystalline ingots of TbCoSi_2_ over the temperature range from 223 K to 298 K. The significant in-plane shrinkage was experimentally confirmed to be induced by structural phase transitions. Before this study, TbCoSi_2_ was generally considered to possess a tetragonal structure with the *Cmcm* space group [29]. Based on the temperature dependence of XRD measurements and Rietveld refinements, we identified the presence of a distinct structure with the *Pbcm* space group in TbCoSi_2_ at low temperatures. Further structural analysis revealed that the drastic shrinkage of the a-axis lattice caused by the structural phase transition accounts for the significant in-plane shrinkage of TbCoSi_2_. Results from macroscopic magnetic measurements and first-principles calculations demonstrate that TbCoSi_2_ exhibits an antiferromagnetic structure with G-type below 13.7 K, and the magnetic phase transition is not related to the structural phase transition [36]. This study presents a metal-based material that can be used for thermal expansion regulation, especially suitable for controlling the thermal expansion properties in the uniaxial direction near room temperature.

## 2. Method

TbCoSi_2_ alloy was prepared by arc melting under a high-purity argon atmosphere. After the remelting of 4 times, the ingot was annealed in a vacuum-sealed quartz tube at 1223 K for 5 days. Then the ingot was cooled slowly. The temperature dependence of linear thermal expansion data (Δ*L*/*L*_0_) were obtained by a thermo-dilatometer (DIL 402 Expedis Select, NETZSCH, Selb, Germany) with a heating rate of 5 K/min. Thermal expansion sample was prepared by grinding and polishing arc-melted button ingot. The length of the polished sample along the in-plane direction is 6 mm, which exceeds the resolution limit of the thermo-dilatometer (1 nm). The variable temperature measurements of XRD were collected using an using an x-ray diffractometer (PW 3040-X’Pert Pro, PANalytical, Almelo, Netherlands) with Cu Kα radiation to confirm the crystal structures [37]. The structure refinements for all XRD data were conducted by FULLPROF software (version March 2021) [38]. The macroscopic magnetism of TbCoSi_2_ was measured by a physical property measurement system (PPMS) of Quantum Design (San Diego, CA, USA).

First-principles calculations were conducted using the Vienna Ab initio Simulation Package (VASP 5.4.4) [39] with the projector augmented-wave (PAW) method [40], treating Tb, Co, and Si valence electrons as 4f^9^6s^2^, 3d^7^4s^3^, and 3s^2^3p^2^, respectively, and employing the Perdew–Burke–Ernzerhof (PBE) exchange–correlation functional [41]. To accurately describe the localized 4f orbitals of Tb, the GGA + U approach [42,43] was applied with U = 5 eV [44]. A plane-wave kinetic-energy cutoff of 400 eV and a Monkhorst–Pack [45] k-point spacing of 2π × 0.03 Å^−1^ were used to ensure total energy convergence within 1 meV per atom. The A-type, G-type, and C-type antiferromagnetic configurations were self-consistently calculated using a 2 × 1 × 2 supercell containing 64 atoms, and all self-consistent calculations took into account the spin–orbit coupling (SOC) effect. Based on ab initio molecular dynamics (AIMD) within the *N_p_T* ensemble with a Langevin thermostat [46], the AIMD simulations were carried out within the 64 atoms for TbCoSi_2_. The high-temperature simulations use the initial structure rather than the structures after the low-temperature simulation. We used a step size of 1 fs during the molecular dynamic simulation.

## 3. Results and Discussion

**Figure 1:**
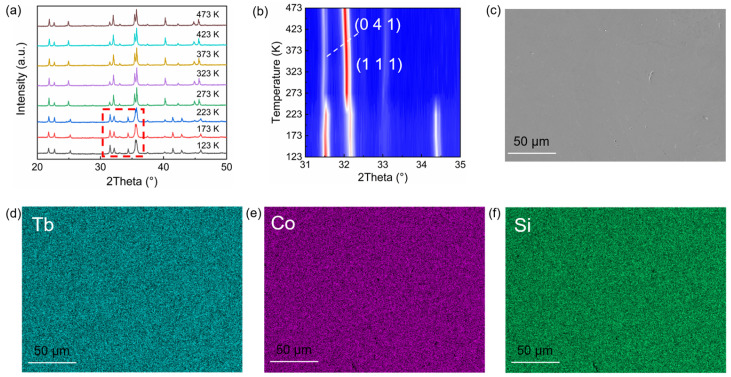
Crystal structure and surface morphology of TbCoSi_2_. (**a**) Temperature dependence of XRD patterns. The diffraction peaks within the red frame exhibit distinct characteristics of a phase transition. (**b**) Temperature dependence of XRD patterns of (041) and (111) peaks. Scanning electron microscope image (**c**) of TbCoSi_2_ and corresponding energy dispersive spectrometer patterns of (**d**) Tb, (**e**) Co, and (**f**) Si.

TbCoSi_2_ exhibits a crystal structure characterized by the *Cmcm* space group as per previous literature [47]. Temperature dependence of X-ray diffraction (XRD) measurements was conducted to probe structural transition across a broad thermal range (Figure 1a). For temperatures above 273 K, most diffraction peaks in the XRD patterns of TbCoSi_2_ exhibit excellent consistency with the standard diffraction profile of TbCoSi_2_ with the *Cmcm* space group, confirming the high-temperature structure. The diffraction peaks at 37° originate from a trace amount of the TbCo_2_Si_2_ secondary phase. Notably, when the temperature is decreased below 273 K, both the number and relative intensities of diffraction peaks undergo significant modifications (highlighted by the red box in Figure 1a). Therefore, the crystal structure below 273 K is distinct from the high-temperature *Cmcm* phase. To clearly capture the variations in diffraction peaks and the associated structural transition, the temperature dependence of the diffraction peak intensities corresponding to the (041) and (111) lattice planes was analyzed in detail (Figure 1b). With the temperature decreasing below 273 K, the intensity of the (041) diffraction peak rises abruptly, whereas that of the (111) diffraction peak exhibits a distinct decrease. Furthermore, a new diffraction peak emerges at an angular position of ~34.4°. Collectively, these observations provide unambiguous evidence for a structural phase transition in TbCoSi_2_ upon cooling, which may be associated with subtle changes in lattice symmetry.

The surface microstructures of arc-melted polycrystalline TbCoSi_2_ were characterized using a scanning electron microscope (SEM, Gemini 500, ZEISS, Oberkochen, Germany). As presented in Figure 1c, the as-synthesized TbCoSi_2_ sample exhibits a highly dense microstructure across its surface. Such structural uniformity is crucial in subsequent property characterizations. To further validate the elemental composition and spatial homogeneity of the TbCoSi_2_ polycrystal, energy-dispersive X-ray spectroscopy (EDS) mapping was performed in conjunction with SEM to enable the visualization of elemental distribution. The elemental distribution maps corresponding to Tb, Co, and Si are presented in Figure 1d, Figure 1e and Figure 1f, respectively. These maps reveal a uniform spatial distribution of all constituent elements throughout the sampled area, with no regions of elemental enrichment or depletion.

**Figure 2:**
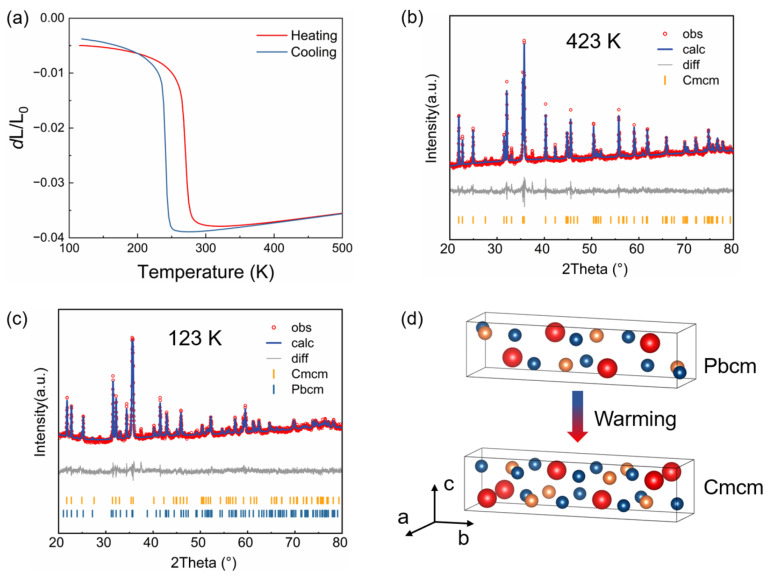
Thermal expansion and Rietveld refinements of TbCoSi_2_. (**a**) Temperature dependence of linear thermal expansion Δ*L*/*L*_0_. (**b**) Rietveld refinement plots for the structure with *Cmcm* space group at 423 K. (**c**) Rietveld refinement plots for the structure with *Cmcm* and *Pbcm* space groups at 123 K. (**d**) Schematic diagram of structural phase transition, red, blue, and gold spheres denote Tb, Co, and Si atoms, respectively.

The thermal expansion and crystallographic structure were characterized by a thermo-dilatometer and Rietveld refinement of XRD patterns. The temperature dependence of linear thermal expansion (Δ*L*/*L*_0_) demonstrates a distinct sharp contraction in TbCoSi_2_ along the in-plane direction at *T*_t_ = 273 K during the heating process (Figure 2a). A corresponding expansion is observed at 250 K during the cooling cycle, which confirms a thermal hysteresis of approximately 25 K. The reversibility and the thermal hysteresis of this phase transition are consistent with its classification as a first-order phase transition. Over the temperature range from 223 K to 298 K, the TbCoSi_2_ ingot exhibits a remarkable contraction of ~3% along the in-plane direction. This value exceeds the linear contraction rate in prominent giant negative thermal expansion materials, including MnCo_0.98_Cr_0.02_Ge (1.3%), Ni_55.5_Mn_19.5_Ga_25_ (0.45%), LaFe_11.5_Si_1.5_ (0.35%), and Hf_0.8_Ta_0.2_Fe_2_ (0.34%) [24,27,48,49,50]. Therefore, TbCoSi_2_ displays an anomalously giant negative thermal expansion in the in-plane direction of the ingot. Such exceptional NTE behavior renders it a highly promising candidate for applications requiring tunable thermal expansion (especially for precise composite materials that need to eliminate thermal mismatch in a single direction) or a phase-transition-driven device.

To elucidate the fundamental physical mechanisms governing the observed thermal expansion anomalies in TbCoSi_2_, Rietveld refinement was systematically performed on the temperature dependence of XRD patterns. As illustrated in Figure 2b, the Rietveld refinement confirms that TbCoSi_2_ adopts a single-phase structure with the *Cmcm* space group at 423 K. The unresolved peaks in the XRD pattern are attributed to trace amounts of TbCo_2_Si_2_ and terbium oxide impurities. As mentioned above, the temperature dependence of XRD patterns indicates that TbCoSi_2_ exhibits a structural transition below 273 K. As presented in Figure 2c, the Rietveld refinement results reveal the coexistence of two distinct phases within TbCoSi_2_ at 123 K. The second phase was identified as belonging to the *Pbcm* space group at low temperature. As shown in Figure 2d, each unit cell of the *Cmcm* phase and *Pbcm* phase of TbCoSi_2_. For the *Cmcm* phase, the lattice parameters are a = 4.02 Å, b = 16.30 Å, c = 3.97 Å, with Wyckoff positions Tb: 4c, Co: 4c, and Si: 8c. For the *Pbcm* phase, the parameters are a = 4.21 Å, b = 15.61 Å, c = 3.95 Å, and Wyckoff positions Tb: 4d, Co: 4d, and Si: 8d. Therefore, the reversible structural phase transition involves a structural transformation between the low-temperature *Pbcm* phase and the high-temperature *Cmcm* phase (Figure 2d), which is the primary driver of the pronounced in-plane contraction observed in the TbCoSi_2_ ingot.

**Figure 3:**
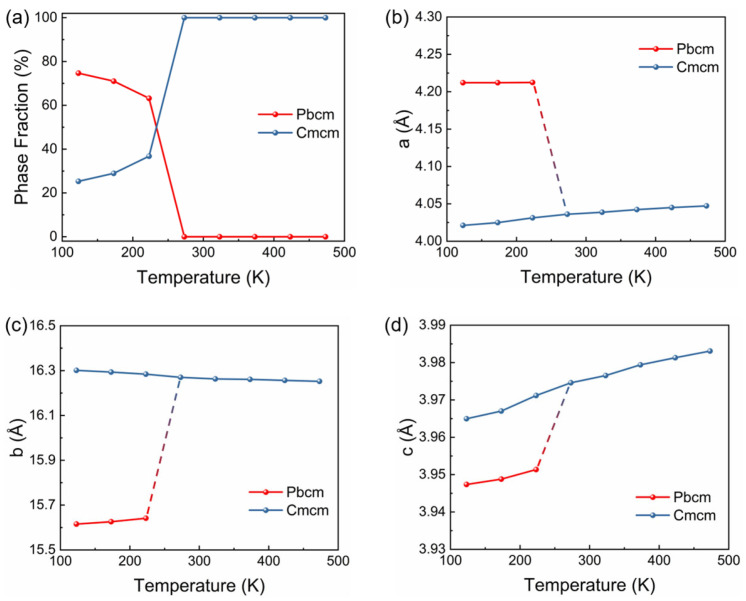
Rietveld refinement results of TbCoSi_2_. (**a**) Temperature dependence of phase fraction of the *Pbcm* and *Cmcm* phases. (**b**–**d**) Temperature dependence of lattice parameters of different crystal axes.

In order to gain deeper mechanistic insight into the in-plane contraction induced by the phase transition, the temperature dependence of phase fractions and the lattice parameters for both the *Cmcm* and *Pbcm* phases were extracted. As shown in Figure 3a, the fraction of the low-temperature *Pbcm* phase reaches as high as 75% at 123K and decreases monotonically with increasing temperature; meanwhile, the fraction of the high-temperature *Cmcm* phase increases. The *Pbcm* phase fraction reaches 0% at 273 K, indicating complete transformation to the *Cmcm* phase. Given that both the *Pbcm* and *Cmcm* phases exhibit tetragonal symmetry, the temperature dependences of the lattice parameters were compared to identify the axis driving the in-plane contraction (Figure 3b–d). From 223 K to 273 K, the a-axis undergoes a significant contraction up to 4.5%, whereas the b-axis and c-axis expand by 4% and 0.5%, respectively. The prominent anisotropy demonstrates that the in-plane contraction is primarily driven by the a-axis. For typical polycrystalline compounds, thermal expansion behavior reflects the average response of all lattice axes. However, the arc-melted TbCoSi_2_ intermetallic compound exhibits significant crystallographic preferred orientation, which originates from the rapid cooling during solidification [3,4]. Consequently, the a-axis of TbCoSi_2_ is predominantly aligned along the plane direction of the ingot, which gives rise to the observed significant in-plane contraction.

**Figure 4:**
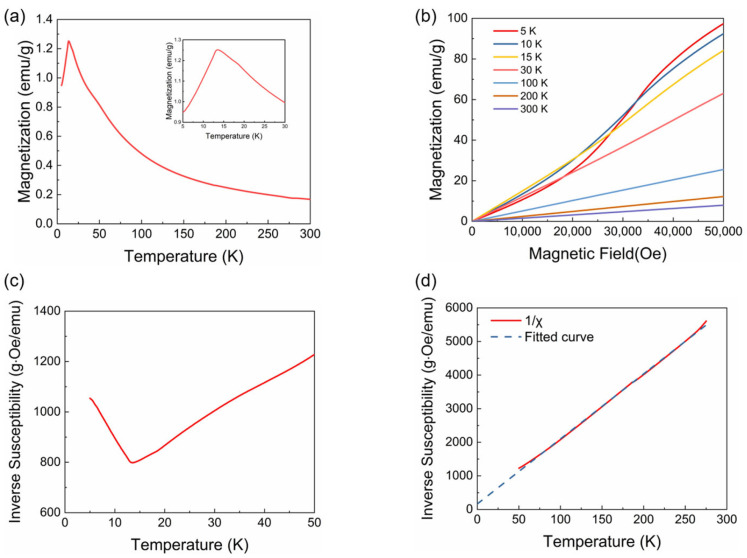
Macroscopic magnetic characteristics of TbCoSi_2_. (**a**) Field-cooling temperature dependence of magnetization (*M*-*T* curve) at a magnetic field of 1000 Oe. (**b**) Magnetic field dependence of magnetization (*M*-*H* curves) at different temperatures. (**c**) Temperature dependence of inverse susceptibility of TbCoSi_2_ from 5 K to 50 K. (**d**) Temperature dependence of inverse susceptibility of TbCoSi_2_ from 50 K to 275 K and corresponding fitted curve.

The magnetic structure of TbCoSi_2_ was characterized by macroscopic magnetic measurements and first-principles calculations. As illustrated in the field-cooling (FC) temperature dependence of the magnetization (*M*-*T*) curve, the TbCoSi_2_ exhibits antiferromagnetic (AFM) ordering at low temperatures, with a Neel temperature (*T*_N_) of approximately 13.7 K (Figure 4a). Near room temperature, TbCoSi_2_ adopts a paramagnetic state. Therefore, the structural phase transition is independent of the magnetic phase transition. In order to further elucidate the magnetic structure of TbCoSi_2_ at low temperatures, we measure the magnetic field dependence of magnetization (*M*-*H*). TbCoSi_2_ compound exhibits paramagnetic behavior above 15 K. Notably, a metamagnetic transition emerges under an applied magnetic field of 2 T, indicating that the AFM structure is reoriented into a ferromagnetic (FM) structure upon field application (Figure 4b). The inverse magnetic susceptibility demonstrates the same AFM characteristics as the *M*-*T* curve (Figure 4c). To further investigate the nature of the magnetic ordered state, the inverse magnetic susceptibility in the paramagnetic region was fitted using the Curie–Weiss law (Figure 4d). The derived paramagnetic Curie temperature (*θ*_p_) is −9.6 K, a value close to the *T*_N_ = 13.7 K. This suggests that AFM interactions dominate the magnetic behavior of TbCoSi_2_. The effective magnetic moment (*μ*_eff_) is determined to be 10.1 *μ*_B_, consistent with the theoretical magnetic moment of free Tb^3+^ ions (9.72 *μ*_B_). Based on these findings, the magnetism of TbCoSi_2_ originates primarily from Tb^3+^ ions, whereas Co ions adopt a low-spin configuration.

**Figure 5:**
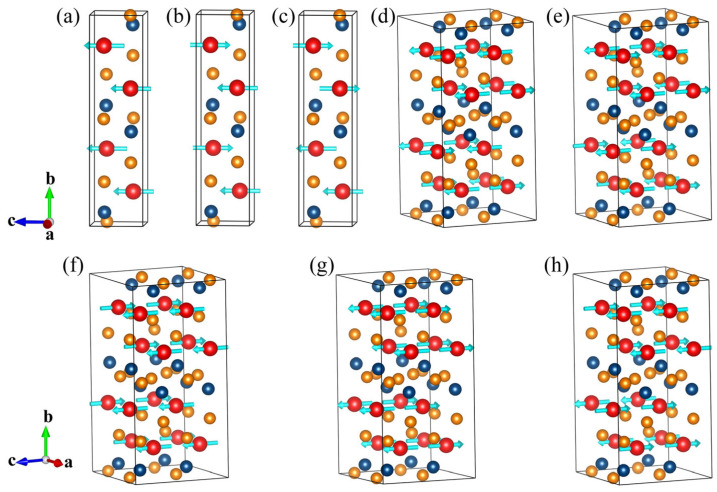
Ferromagnetic and several representative antiferromagnetic configurations of *Pbcm* TbCoSi_2_ along the [001] direction. (**a**) Ferromagnetic configuration: ferromagnetic both within and between the Tb atoms layers; (**b**,**c**) A-type AFM configurations ferromagnetic within the Tb layer and antiferromagnetic coupling between the layers, A_1_ and A_2_; (**d**,**e**) G-type AFM configurations, G_1_ and G_2_, featuring antiferromagnetic coupling both within and between Tb layers; (**f**–**h**) C-type AFM configurations, C_1_, C_2_, and C_3_, exhibiting antiferromagnetic intralayer and ferromagnetic interlayer interactions. The blue arrows on Tb atoms indicate the directions of magnetic moments.

First-principles calculations were performed to investigate the magnetic properties of *Pbcm* TbCoSi_2_ at 0 K, as illustrated in Figure 5. To determine the magnetic ground state, one ferromagnetic configuration (Figure 5a) and three representative antiferromagnetic configurations—A-, C-, and G-types (Figure 5b–h)—were examined. These AFM types were chosen because they are able to describe as closely as possible the fundamental combination of intra- and inter-layer magnetic interactions of the structure, thus enabling a comprehensive description of the possible antiferromagnetic arrangements [51]. In the A-type AFM configuration, all spins within each Tb atomic layer align ferromagnetically to form a magnetic monolayer, while adjacent layers couple antiferromagnetically. In contrast, C-type AFM configurations feature antiferromagnetic ordering within each Tb layer but ferromagnetic coupling between layers, whereas G-type AFM corresponds to antiferromagnetic coupling both within and between Tb layers. For each type, spin orientations along the [100], [010], and [001] crystallographic directions were considered, yielding a total of 24 distinct magnetic configurations. Representative models are shown in Figure 5, including the A_1_ and A_2_ variants of the A-AFM (Figure 5b,c), the G_1_ and G_2_ variants of the G-AFM (Figure 5d,e), and the C_1_, C_2_, and C_3_ variants of the C-AFM (Figure 5f–h) The calculations reveal that variations in magnetic ordering have a negligible influence on the lattice volume, meaning that energy differences can reliably replace enthalpy differences in subsequent analysis. A direct comparison of total energies shows that the FM configuration is always higher in energy than AFM configuration, indicating that the *Pbcm* TbCoSi_2_ is intrinsically antiferromagnetic, in agreement with experimental results. Among the AFM configurations, the G_2_ type with spin orientation along the [001] direction is the most stable, with a lower energy than other AFM states. This result suggests that the *Pbcm* phase stabilizes in the G_2_-type AFM configuration with spins preferentially aligned along the crystallographic c-axis.

Further analysis elucidates the relative significance of various exchange interactions. In all A-, C-, and G-type AFM configurations, the magnetic moments preferentially lie within the basal plane, consistent with pronounced in-plane magnetic anisotropy. The energy difference between the G_1_- and G_2_-AFM variants along the [001] direction is less than 0.6 meV/atom, indicating that the next-nearest-neighbor exchange interaction is negligible. In contrast, the energy variation of the G_1_-AFM configuration among the [001], [010], and [100] directions reaches up to 2.1 meV/atom, revealing substantial anisotropy in the AFM coupling. This pronounced anisotropy originates from the strong spin–orbit coupling inherent to heavy Tb atoms, where spin–orbital interactions align the magnetic moments along specific crystallographic axes, leading to notable direction-dependent SOC energies. Moreover, the small energy difference between the C-type and G-type AFM states suggests that when intralayer AFM order dominates, interlayer exchange interactions contribute only weakly. Collectively, these findings demonstrate that the fundamental magnetic behavior of *Pbcm* TbCoSi_2_ is primarily governed by the interactions within the Tb layer and strong self-consistent spin–orbital coupling. In contrast, interlayer interactions play secondary roles. This insight provides a foundation for understanding its intrinsic AFM ground state of TbCoSi_2_.

**Figure 6:**
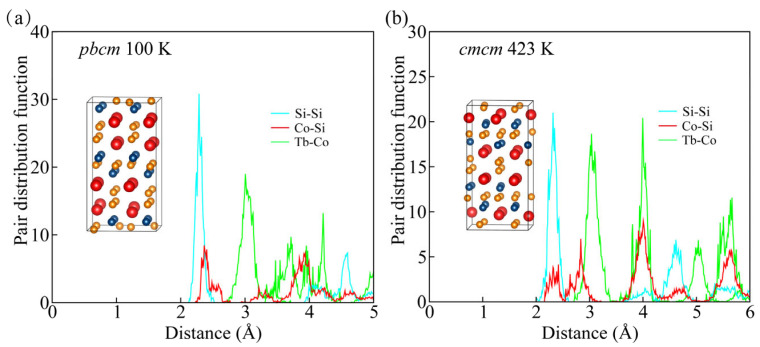
Thermal stability analysis of TbCoSi_2_. Pair distribution functions for the *Pbcm* phase of TbCoSi_2_ at 100 K (**a**) and the *Cmcm* phase of TbCoSi_2_ at 423 K (**b**), respectively. The inset depicts the terminal structure.

To evaluate the thermal stability of TbCoSi_2_, ab initio molecular dynamics (AIMD) simulations were performed for both the low-temperature and high-temperature phases. The equilibrium structures were obtained from the final configurations of the respective AIMD simulations. It is noted that the high-temperature simulation was initialized from the corresponding experimental structure, rather than the configuration obtained after the low-temperature simulation. As depicted in Figure 6a and Figure 4b, the *Pbcm* phases of TbCoSi_2_ at 100 K and *Cmcm* phase of TbCoSi_2_ 423 K, respectively. The first peaks in the pair distribution functions reveal that the nearest Si–Si, Co–Si, and Tb–Co distances in the equilibrium structures show negligible deviation from those in the initial configurations, indicating excellent thermal stability. These results demonstrate that the Pbcm phase remains stable at low temperature, whereas the *Cmcm* phase is stable at high temperature, in good agreement with the experimental observations.

## 4. Conclusions

Insights into the structure phase transition in TbCoSi_2_ reveal the mechanism underlying the giant in-plane shrinkage behavior of TbCoSi_2_ polycrystalline ingots, steering the discovery and application of new weakly magnetic NTE materials. Temperature dependence of XRD and Rietveld refinements confirms the new crystal structure of the *Pbcm* space group at low temperature. Analysis of the extracted lattice constant indicates that the contraction along the a-axis during the phase transition is the primary driver of the in-plane shrinkage of the TbCoSi_2_ ingot. Macroscopic magnetic measurements, Curie-Weiss fitting, and first-principles calculations demonstrate that TbCoSi_2_ exhibits an antiferromagnetic ground state below 13.7 K, which is far below the working temperature range of NTE. These findings not only provide a comprehensive understanding of the crystal structure and magnetic structure of TbCoSi_2_ but also offer a metal-based weakly magnetic NTE material that exhibits a significant in-plane contraction near room temperature.

## Data Availability

The original contributions presented in this study are included in the article. Further inquiries can be directed to the corresponding author.
